# The WRKY Transcription Factor Genes in *Lotus japonicus*

**DOI:** 10.1155/2014/420128

**Published:** 2014-03-16

**Authors:** Hui Song, Pengfei Wang, Zhibiao Nan, Xingjun Wang

**Affiliations:** ^1^College of Pastoral Agriculture Science and Technology, Lanzhou University, Lanzhou 730000, China; ^2^Bio-Tech Research Center, Shandong Academy of Agricultural Sciences, Shandong Provincial Key Laboratory of Crop Genetic Improvement, Ecology and Physiology, Jinan 250100, China

## Abstract

WRKY transcription factor genes play critical roles in plant growth and development, as well as stress responses. WRKY genes have been examined in various higher plants, but they have not been characterized in *Lotus japonicus*. The recent release of the *L. japonicus* whole genome sequence provides an opportunity for a genome wide analysis of WRKY genes in this species. In this study, we identified 61 WRKY genes in the *L. japonicus* genome. Based on the WRKY protein structure, *L. japonicus* WRKY (LjWRKY) genes can be classified into three groups (I–III). Investigations of gene copy number and gene clusters indicate that only one gene duplication event occurred on chromosome 4 and no clustered genes were detected on chromosomes 3 or 6. Researchers previously believed that group II and III WRKY domains were derived from the C-terminal WRKY domain of group I. Our results suggest that some WRKY genes in group II originated from the N-terminal domain of group I WRKY genes. Additional evidence to support this hypothesis was obtained by *Medicago truncatula* WRKY (MtWRKY) protein motif analysis. We found that LjWRKY and MtWRKY group III genes are under purifying selection, suggesting that WRKY genes will become increasingly structured and functionally conserved.

## 1. Introduction

Transcription factors are crucial in regulating gene expression. Transcription factors present sequence-specific DNA binding sites and are able to modulate the transcription rate of downstream target genes [1]. WRKY genes have primarily been located in plants, where they are one of the most important transcription factor families [2]. WRKY genes are defined by having a unique WRKY domain of approximately 60 amino acid residues [2]. The WRKY domain contains a highly conserved amino acid sequence WRKYGQK at the N-terminal and a metal chelating zinc finger motif (C–X_4-5_–C–X_22-23_–H–X–H, (C_2_H_2_) or C–X_5–8_–C–X_25–28_–H–X_1-2_–C, (C_2_HXC)) at the C-terminal end [2–5]. In some WRKY genes, the WRKY domain can be characterized as WRRY, WSKY, WKRY, WVKY, or WKKY [6]. WRKY transcription factors interact with the W-box (TTGAC[T/C]) sequence in promoter regions to modulate gene expression [4, 7, 8]. In addition, WRKY transcription factors bind SURE, a novel* cis*-element in higher plants, to regulate sugar response [9].

Members of the WRKY family can be classified into three groups according to the number of WRKY domains and the pattern of the zinc finger motif [2]. Generally, group I WRKY transcription factors contain two WRKY domains with distinct functions. Previous studies have demonstrated that the C-terminal WRKY domain mediates sequence-specific binding to the target DNA [8, 10, 11]. It has been proposed that the N-terminal WRKY domain increases the affinity or specificity of these proteins to the target sites [2]. Group II and III WRKY transcription factors contain one WRKY domain with a C_2_H_2_ zinc finger motif and C_2_HXC zinc finger motif [2]. Based on a phylogenetic analysis of the WRKY family, the members of group II can be divided into five subgroups: IIa, IIb, IIc, IId, and IIe [2].

Since the cloning a WRKY gene cDNA from* Ipomoea batatas* [11], a large number of WRKY protein genes have been cloned from different plant species [3, 4, 12–24]. So far, only two WRKY homologues have been identified from nonplant species,* Giardia lamblia* [5] and* Dictyostelium discoideum* [25].

Plant WRKY genes regulate plant growth and development under normal and stressful conditions [26, 27]. Early studies found that WRKY genes play an important role in gene expression responses to sucrose [11]. Studies in* Arabidopsis* [17, 28, 29], rice [30], tobacco [31, 32], and parsley [33] have indicated that WRKY proteins play key roles in plant responses to pathogens [27, 34]. In addition, previous studies revealed the involvement of WRKY proteins in abiotic stress responses [27], for example, to high temperatures [35], low temperatures [13], salt and drought [24], H_2_O_2_ [36], and UV radiation [37]. WRKY proteins have also been reported to upregulate in response to herbivory [38], nematode damage [39], and wounding [40]. Moreover, WRKY genes may be involved in seed development [9, 41], dormancy and germination [42–44], plant senescence [45, 46] and regulation of metabolic pathways [9], trichome morphogenesis [47], and plant growth [48].

*Lotus japonicus*, an important forage crop in the legume family, is planted in many parts of the world. It has been used extensively in plant research as a model legume, due to its short life cycle (2-3 months), self-fertility, and relatively simple diploid genome [49]. Since the release of the whole genome sequence of* L. japonicus*, it is now possible to compare transcription factors in this plant with other plants. In this study, we analyzed 61 putative WRKY genes from the* L. japonicus* genome. We conducted a phylogenetic analysis to evaluate gene duplications, chromosomal localization, motif analysis, gene structure, and selection pressure analysis of group III WRKY genes to provide information about WRKY gene family evolution in* L. japonicus.*

## 2. Materials and Methods

### 2.1. Sequence Database Search

The* L. japonicus* genome sequence (build 2.5) was downloaded from http://www.kazusa.or.jp/lotus/. The complete set of WRKY gene sequences was identified using a deliberative process. First, a Hidden Markov Model (HMM) profile of the WRKY domain (PF03106) was downloaded from the Pfam database (http://pfam.sanger.ac.uk/) [50]. We employed the WRKY domain as a query to identify all possible WRKY gene sequences in the* L. japonicus* genome database using the BLASTp program (*P* value = 0.001). Subsequently, a search on the Pfam database was used to confirm and classify each putative WRKY sequence. We located overlapping genes by aligning all of the candidate WRKY gene sequences using Clustal W [51]. Only the nonoverlapping WRKY sequences were used for further analysis.

### 2.2. Gene Duplication and Chromosomal Locations of WRKY Genes

To detect potential gene duplications, we aligned and calculated all of the relevant genes identified in* L. japonicus* genomes. We defined gene duplication between any two loci such that [52]: (1) the alignable nucleotide sequence covered >70% of the longer sequence; (2) the amino acid identity between the sequences was >70% identical.

In order to determine the physical locations of WRKY genes in chromosomes, we blasted each WRKY gene as a query against the* L. japonicus* genome (http://www.kazusa.or.jp/lotus/) to determine the initiation site of each gene. MapInspect software was used to draw the location images of the WRKY genes (http://www.plantbreeding.wur.nl/uk/software_mapinspect.html).

### 2.3. Multiple Sequence Alignments, Phylogenetic, and Gene Structure Analysis

To examine the domain organization of WRKY proteins in detail, multiple sequence alignments of WRKY domain sequences were performed using Clustal W [51]. Phylogenetic and molecular evolution analyses of WRKY proteins were conducted in MEGA 4.0 [53]. Phylogenetic trees were estimated with the Neighbor-Joining method. Bootstrap analyses of 1,000 repetitions were obtained for each tree to analyze statistical support for nodes. The complete amino acid sequences of group III WRKY genes were analyzed for evidence of selection pressure [54]. Gene structure display server (GSDS) software [55] was used to illustrate exon-intron organization for individual WRKY genes by comparing the cDNA sequence with the corresponding genomic DNA sequence.

### 2.4. Identification of Conserved Motifs

The program MEME 4.9 [56] was used to predict motifs in the WRKY domain with the following parameters: (1) any number of repetitions, (2) an optimum motif width between 6 and 200 residues, (3) and a maximum of 20 motifs. Structural motif annotation was performed using the Pfam database.

### 2.5. Selection Pressure in Group III WRKY Proteins

The amino acid sequences of group III* Medicago truncatula* (MtWRKY) and* L. japonicus* WRKY (LjWRKY) proteins were used to estimate phylogenetic trees and then the trees were used to detect evidence of selection. The PAL2NAL program [57] was used for conversion of a protein sequence into the corresponding nucleotide sequence. We used PAML 4.7 [58] to analyze codon substitution patterns in a maximum likelihood framework, implementing a site-specific model. The program CODEML was employed to calculate the *d*_N_/*d*_S_ ratio (or *ω*), the ratio of nonsynonymous/synonymous distances. Generally, *ω* = 1, >1, and <1 indicate neutral, positive, and purifying selection, respectively. We detected variation in *ω* among sites by employing a likelihood ratio test (LRT) between M0 versus M3 and M7 versus M8 [54]. The nodes were considered to have undergone positive selection, if they satisfied the following criteria [59]: (1) an estimate of *ω* > 1 under M8, (2) sites identified to be under positive selection by Bayes Empirical Bayes (BEB) analysis, (3) and a statistically significant LRT.

## 3. Results

### 3.1. Identification and Classification of WRKY Genes

In this study, a total of 71 WRKY genes in the* L. japonicus* genome (build 2.5) were identified (Table 1). Among these sequences, 10 WRKY genes were excluded from this study due to the divergent structures of the putative proteins in these genes, a lack of specific domains or motifs, and the short length of the WRKY domain, generally 2/3 of the normal WRKY domain length. Although the LjWRKY51 protein, including two WRKY domains, lacked a complete WRKY domain at the C-terminal region, this protein was retained for subsequent analyses.

**Figure 1:**
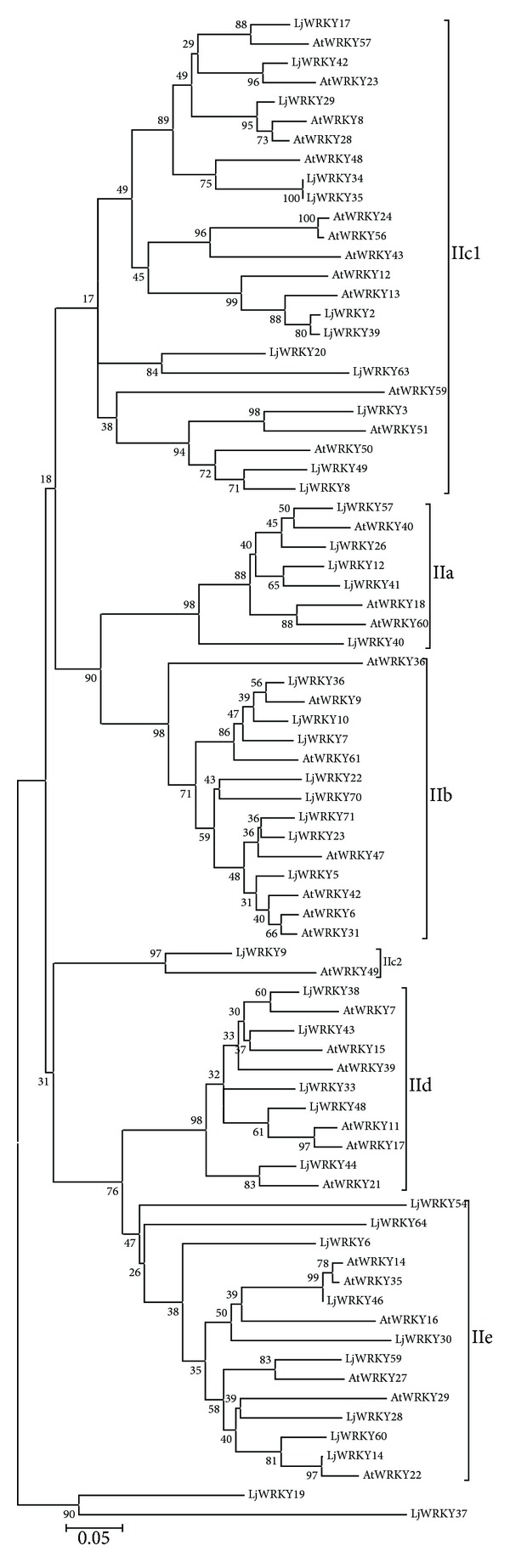
Phylogenetic tree of* Arabidopsis* WRLY and* Lotus japonicus* WRKY domain. The phylogenetic tree was constructed using MEGA 4.0 by the Neighbor-Joining (NJ) method with 1,000 bootstrap replicates. The percentage bootstrap scores higher than 50% are indicated on the nodes. The AtWRKY domains from Eulgem et al. (2000).

Among these 61 WRKY genes, there were 12 group I WRKY genes, 42 group II WRKY genes, and 7 group III WRKY genes, based on the number of WRKY domains and the type of zinc finger motifs (Tables 1 and 2). To obtain a better classification within group II WRKY proteins, we constructed a phylogenetic tree with 42 group II LjWRKY using the* Arabidopsis* WRKY sequence as a reference [2] (Figure 1). It was found that LjWRKY genes in group II could be divided into six subgroups, including 5 members in subgroup IIa, 8 in subgroup IIb, 13 in subgroup IIc, 5 in subgroup IId, 9 in subgroup IIe, and 2 in subgroup IIx (Tables 1 and 2 and Figure 1). Among them, LjWRLY19 and LjWRKY37 genes did not cluster with* Arabidopsis*, and we temporarily named this group IIx (Figure 1).

We found 9 potential pseudogenes among 61 WRKY genes, with either a premature stop codon or a frame shift mutation. Among these pseudogenes, 3 genes were found in group I, 2 in group IIa, 2 in group IIc, and 2 in groups IIx and IIe (Table 1).

### 3.2. Gene Duplication and Chromosomal Locations of WRKY Genes

**Figure 2:**
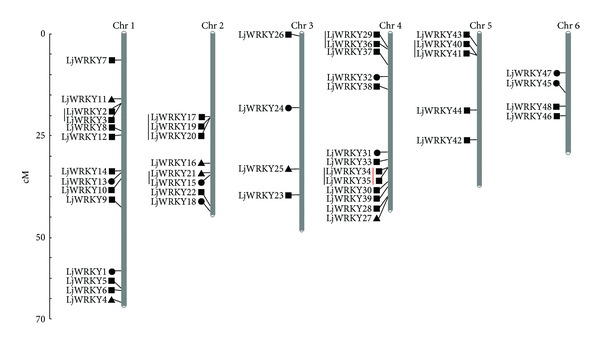
Chromosomal locations of* Lotus japonicus* WRKY genes. The chromosome numbers were shown at the top of each chromosome (chromosome; gray bars). The names on the left side of each chromosome correspond to the approximate location of each WRKY gene. The markers next to the gene names represented the groups to which each WRKY gene belongs (●: group I; ■: group II; ▲: group III). The black lines on the left side of the names of the WRKY genes indicated the clusters of gene on each chromosome. The red line on the left side of the markers indicated the duplicated genes. Unmapped WRKY genes were not shown.

Gene duplication, including tandem and segmental duplication events, plays a crucial role in genomic expansions. Two or more duplicated genes located in the same chromosome are defined as tandem duplication, while other types of gene duplication are defined as segmental duplication events. We detected only one tandem duplication (LjWRKY34 and LjWRKY35) on chromosome 4 (Figure 2), suggesting that WRKY genes in* L. japonicus* are not recently generated by gene duplication.

A total of 48 WRKY genes could be mapped to chromosomes 1–6, and the others could not be conclusively mapped to any chromosomes, because some WRKY genes have no precise location information. Fourteen WRKY genes including two group I, 10 group II, and two group III genes were located on chromosome 1. Thirteen genes were mapped to chromosome 4. Four genes were mapped to chromosome 3 (one group I, two group II, and one group III genes) and chromosome 6 (two group I and two group II genes), respectively. Eight (two group I, four group II, and two group III genes) and five WRKY genes (five group II genes) were found on chromosome 2 and chromosome 5, respectively (Figure 2).

A gene cluster is defined as a chromosome region with two or more genes located within 200 kb sequence [60]. Using this criterion, we found 13 WRKY genes forming six gene clusters. Chromosomes 2 and 4 each contain two gene clusters, while only one gene cluster was found on chromosomes 1 and 5, respectively (Figure 2). No clusters were found on chromosomes 3 and 6. Through chromosomal location and gene cluster analysis, we found that the number of genes on chromosomes is disproportionate to the number of gene clusters. For example, 14 WRKY genes were found on chromosome 1, which contains only one gene cluster.

### 3.3. Phylogenetic Analysis and Gene Structure

**Figure 3:**
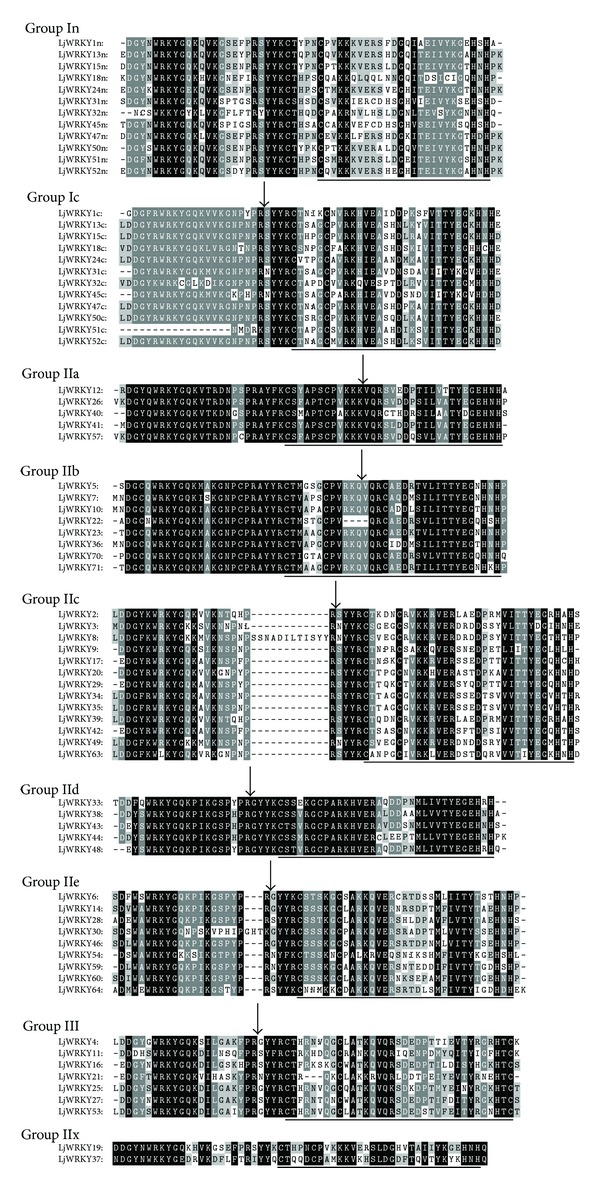
Amino acid residue alignment of* Lotus japonicus* WRKY domain. Alignment was performed using the Clustal W program and is displayed with the software Gendoc. Residues that were highly conserved within each of the major groups are in black. The position of a conserved intron was indicated by an arrowhead. The black lines indicated the conserved zinc finger motifs.

Amino acid residues of WRKYGQK are the distinguishing regions of the WRKY transcription factor [2, 27]. Multiple alignment analysis of LjWRKY domains found that mutations occurred at R, Y, and Q in the conserved WRKYGQK sequence (Figure 3). Further study showed that the variation arose from amino acid substitutions of R to K or L or from K to C and from Q to Y, E, L, or K (Figure 3). On the other hand, we identified a CX_4_CX_22_HXH zinc finger motif in subgroup In and IIx genes, a CX_4_CX_23_HXH motif in subgroup Ic and IIe genes, and a CX_5_CX_23_HXH motif in subgroup IIa, IIc, IId, and IIe genes (Figure 3). We found that the WRKY domain was replaced by WKKY in subgroup In and IIx genes, suggesting that subgroup IIx genes originated from N-terminal WRKY domain of group I genes. The subgroup IIa and IIe genes seem to have formed after the group I genes lost alternative WRKY domains.

**Figure 4:**
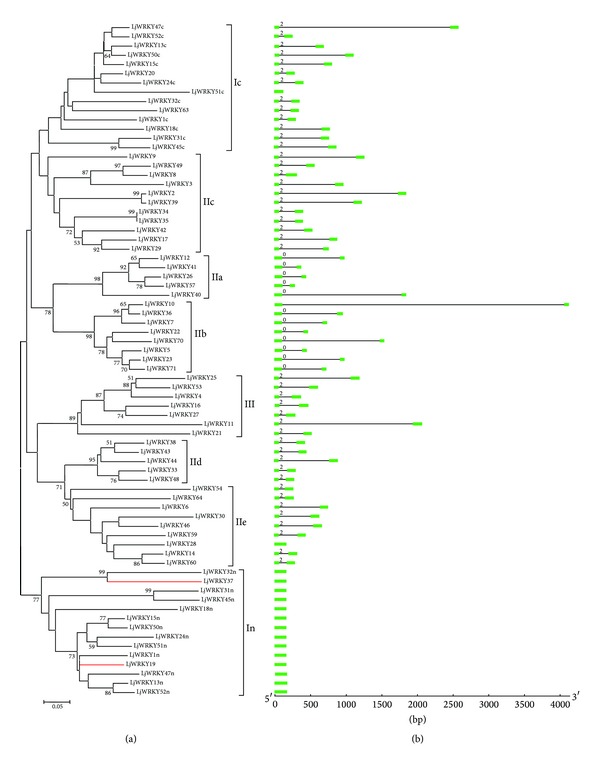
Phylogenetic relationships and gene structure of* Lotus japonicus* WRKY domains. (a) Multiple alignments of WRKY domain amino acids executed by Clustal W and the phylogenetic tree constructed using MEGA 4.0 by the Neighbor-Joining (NJ) method with 1,000 bootstrap replicates. The red line indicated group IIx member. The percentage bootstrap scores higher than 50% were indicated on the nodes. (b) Exon-intron structures of WRKY domain genes from* Lotus japonicus*. Exons and introns were represented by green boxes and black lines, respectively. The number indicated introns phases. The sizes of exons and introns can be estimated using the scale at the bottom.

The WRKY domain phylogenetic tree can be subdivided into eight clades: In, Ic, IIa, IIb, IIc, IId, IIe, and III (IIx nested in In; Figure 4(a)). The group I proteins contain two WRKY domains located at the N-terminal domain (In) or the C-terminal domain (Ic). Although In and Ic belong to group I, members of In and Ic were clustered in different clades, representing the sequence divergence of WRKY domain in In and Ic.

Clade Ic contained 14 members in* L*.* japonicus*, including 12 members with WRKY domains at the C-terminal region and two group II members (LjWRKY20 and LjWRKY63) (Figure 4(a)). These two group II members were clustered with LjWRKY24c and LjWRKY32c, respectively, indicating a common origin of their domains. Moreover, two group II members (LjWRKY19 and LjWRKY37) were also found in the In clade.

Group II can be divided into five clades with high bootstrap values (Figure 4(a)). Further analysis revealed that IIa and IIb form a single clade and IId and IIe form a clade, while IIc contained 11 LjWRKY members form a clade (Figure 4(a)). This suggests that the domains had a recent gene ancestor or formed under similar selective pressures. Clade III contained seven members which are more similar to clade IId and clade IIe than any other members (Figure 4(a)), suggesting that they may have shared an ancestor before divergence of group II and group III.

During analysis of the cDNA and DNA sequences, we found that most of the LjWRKY genes contained two types of introns in their WRKY domains. The phase-2 intron is spliced exactly after the R position, similar to the splicing position observed in* Arabidopsis* [2]. We designate the phase-2 intron as an R-type intron. A phase-0 intron is located before the V position, at the sixth amino acid after the second C residue in the C_2_H_2_ zinc finger motif [22]. We designate the phase-0 intron as a V-type intron. Interestingly, in subgroups Ic, IIc, IId, IIe, and III, the R-type intron is located before the zinc finger motif region in the WRKY domain of genes, while in subgroups IIa and IIb, the V-type intron is found within the zinc finger motif region in the WRKY domain (Figure 4(b)). Furthermore, introns have been lost from LjWRKY28 (subgroup IIe), LjWRKY51c (subgroup Ic), and subgroups In and IIx (Figure 4(b)). Intron loss can be considered as the result of intron turnover, the result of homologous recombination between an intron-containing allele and a mature mRNA [22].

### 3.4. Conserved Motifs in LjWRKY Proteins

**Figure 5:**
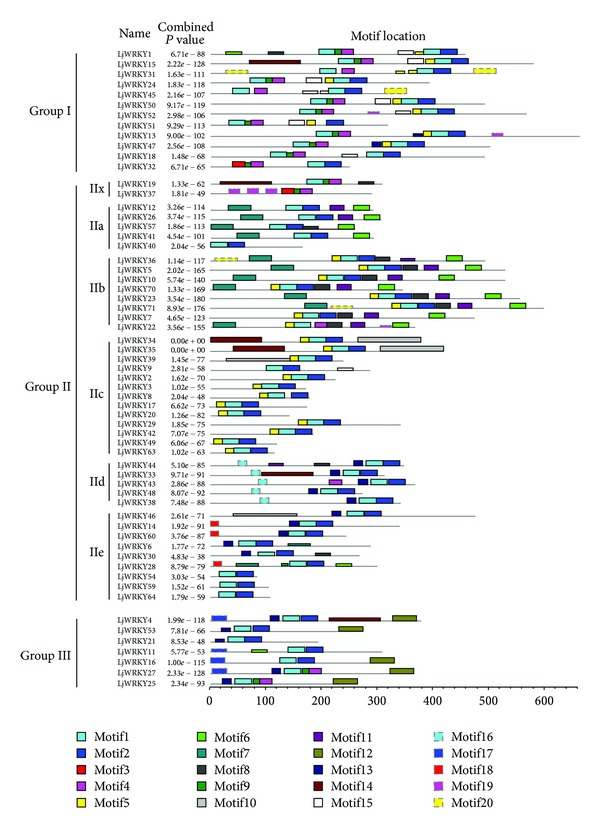
Distribution of 20 predicted conserved motifs in* Lotus japonicus* WRKY proteins. Motifs of* Lotus japonicus* WRKY proteins were identified by MEME program. The conserved amino acid sequences and length of each motif are shown in Table 3.

With the exception of the conserved 60 amino acid residues, no functional or structural homologies were previously known from the remainder of the WRKY protein sequences [2]. Analysis of the 20 motifs revealed that LjWRKY motif lengths ranged from 11 to 113 and the distribution of 20 motifs in each amino acid sequence varied greatly (Table 3 and Figure 5). In addition, the function of the majority of LjWRKY motifs could not be predicted. Unexpectedly, we observed a herpes virus glycoprotein motif (motif14) in LjWRKY34 and LjWRKY35, which has not been reported in previous studies of WRKY genes. It will be interesting to analyze the function of this motif in LjWRKY genes in the future.

Compared with motifs of the WRKY protein from* Arabidopsis* [2],* Populus trichocarpa* [61], and* Oryza sativa* [22], we detected three conserved motifs in LjWRKY genes, including Leu zipper, HARF, and NLS motifs. Subgroup IIa (LjWRKY12, LjWRKY26, LjWRKY41, and LjWRKY51), IIb (LjWRKY5, LjWRKY10, LjWRKY22, LjWRKY23, LjWRKY36, LjWRKY70, and LjWRKY71), and IIe (LjWRKY28) genes contained a Leu zipper motif (motif7). This motif is a hypothetical structure common to a new class of DNA binding proteins. A HARF motif (motif16) was distributed in subgroup IId (LjWRKY33, LjWRKY38, LjWRKY43, LjWRKY44, and LjWRKY48) genes and an NLS motif (motif13) was observed in group I, subgroup IId, subgroup IIe, and group III (LjWRKY13, LjWRKY47, LjWRKY33, LjWRKY38, LjWRKY43, LjWRKY44, LjWRKY48, LjWRKY6, LjWRKY14, LjWRKY30, LjWRKY46, LjWRKY60, LjWRKY4, LjWRKY21, LjWRKY25, LjWRKY27, and LjWRKY53) genes, but their functions are not clear. Previous studies have shown that WRKY proteins contain a coactivator motif (LXXLL or LXLXLX. L, leucine; X, any amino acid), suggesting the role of these motifs in plant immune responses [22, 61]. In this study, we found a probable coactivator motif in group III genes (LjWRKY4, LjWRKY16, LjWRKY25, LjWRKY27, and LjWRKY53), suggesting the involvement of group III genes in response to pathogens.

### 3.5. Evolutionary Analysis of Group III Genes in Plants to Determine Selection Pressure in* L. japonicus* and* M. truncatula*

**Figure 6:**
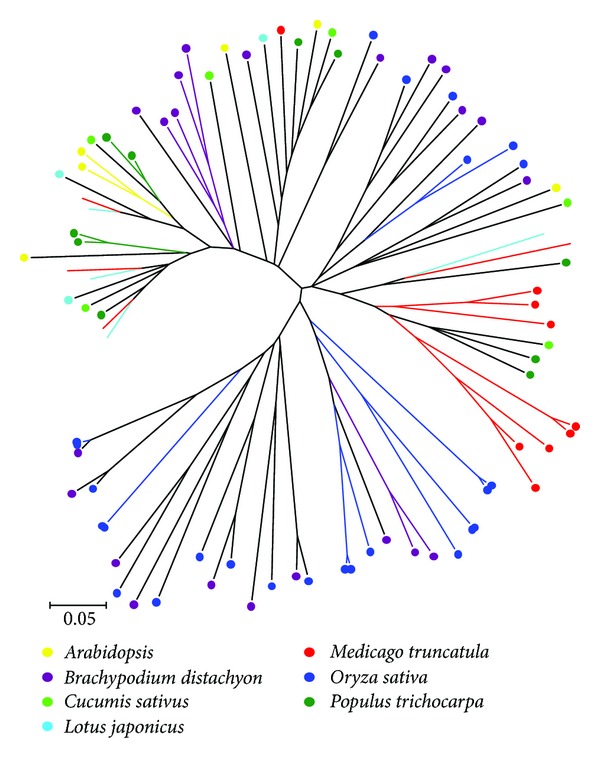
Phylogenetic tree of group III WRKY domains from* Arabidopsis*,* Brachypodium distachyon*,* Cucumis sativus*,* Lotus japonicus*,* Medicago truncatula*,* Oryza sativa*, and* Populus trichocarpa*. The phylogenetic tree was constructed using MEGA 4.0 by the Neighbor-Joining (NJ) method with 1,000 bootstrap replicates. Each WRKY predicted orthologous gene was indicated in a specific color.

In order to study the phylogenetic relationships of group III genes, a phylogenetic tree was estimated using the WRKY domain of seven species, including monocots and dicots. Group III LjWRKY genes did not form a clade; on the contrary, group III LjWRKY genes formed a clade with MtWRKY genes (Figure 6). This suggests that we did not find any paralogs of group III LjWRKY genes, but it suggests that group III LjWRKY genes are orthologous to MtWRKY genes. Paralogous relationships were observed among WRKY genes in other species (i.e., MtWRKY, AtWRKY, PtWRKY, OsWRKY, and BdWRKY). Gene duplication events are considered as the most likely process to result in paralogous copies of genes [59].

**Figure 7:**
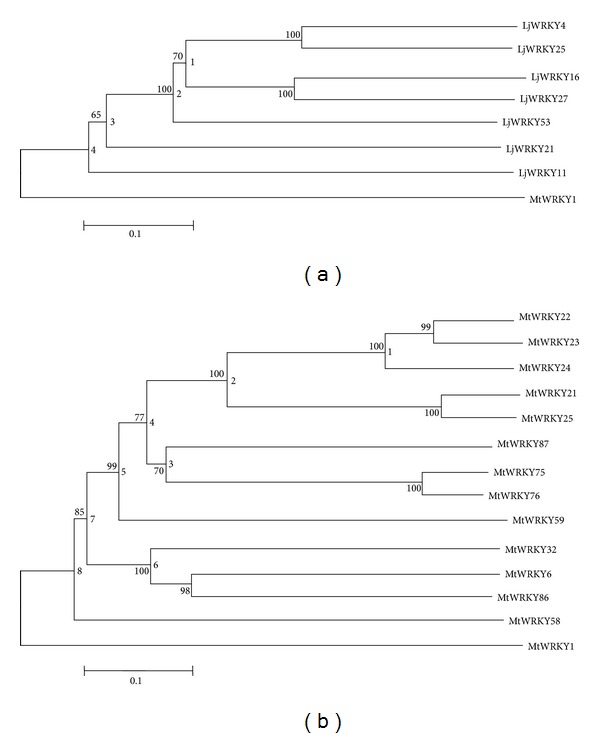
Phylogenetic tree of group III WRKY genes of* Lotus japonicus* and* Medicago truncatula*. The phylogenetic trees were constructed using MEGA 4.0 by the Neighbor-Joining (NJ) method with 1,000 bootstrap replicates. Numbers on the left of each internal node represented bootstrap support values and numbers on the right of each node represented the nodes that were used for positive selection analysis. MtWRKY1 gene was used as outgroup. The tree represented phylogenetic relationships among LjWRKY genes (a) and MtWRKY genes (b).

To detect whether selection pressure affected group III LjWRKY genes, *ω*  (*d*_N_/*d*_S_) was calculated for phylogenetic nodes in PAML (Table 4 and Figure 7). In* L. japonicus* and* M. truncatula*, the ML estimations of *ω* values for all nodes under the model M0 were <1 (Table 4), suggesting that group III LjWRKY and MtWRKY genes have been under purifying selection during evolution. Nevertheless, the log likelihood ratio differences between models M3 and M0 were statistically significant for all nodes tested, except nodes 1 and 2 in MtWRKY (Table 4). This indicates that some genes may be under positive selection. Interestingly, we further analyzed the positive selection in group III genes with models M8 and M7. The *ω* values for all nodes were ≥1 under M8. However, only one node identified one positive selection site in group III MtWRKY genes under model M8 (Table 4). This result shows that group III WRKY genes in* L. japonicus* and* M. truncatula* have not undergone positive selection.

## 4. Discussion

WRKY genes are commonly found in land plants and many WRKY genes have been identified and classified in* Arabidopsis* [2],* Oryza sativa* [6, 22, 62],* Hordeum vulgare* [63],* Cucumis sativus* [59],* Brachypodium distachyon* [64], and* Populus trichocarpa* [61]. However, little information has been reported on WRKY genes in leguminous forage crops. In 2008, approximately 67% of the* L. japonicus* genome (472 Mb) sequences were available on public databases, representing 91.3% coverage of the gene space [49]. In our current work, we conducted an analysis of 61 WRKY genes in the* L. japonicus* genome.

One hundred and four WRKY genes were identified in* P. trichocarpa*, while only 55 WRKY genes were discovered in* C. sativus* and* Vitis vinifera* genomes, respectively (Table 2). The relative number of WRKY genes was not associated with genome size. For example, the number of WRKY genes was about 2x greater in* P. trichocarpa* (104) than in* C. sativus* (55), while these two plants have an approximately equal genome size (458 Mb and 487 Mb; Table 2). By analyzing the number of WRKY gene groups or subgroups (except for subgroup IIx) in each species where they have been characterized, we discovered an uneven distribution of the number of WRKY genes in each group. In rice, the largest number of WRKY genes (36) was found in group III, but only four genes were found to belong to subgroup IIa (Table 2). In* C. sativus*, however, more WRKY genes (16) are classified as subgroup IIc and fewer WRKY genes (4) are categorized in subgroup IIa (Table 2).

Although gene duplication events seem to have led to the expansion of WRKY genes in the* Arabidopsis* [2] and* Oryza* [22] genomes, duplicated WRKY genes were not detected in* C. sativus* [59]. It is not yet clear whether gene duplication typically occurs during LjWRKY gene evolution. Among the 61 WRKY genes, we found that, in* L. japonicus*, only 2 of them were involved in duplication events. In contrast, 11 gene duplication events were identified in the model plant* M. truncatula*. In addition, there were 42 PtWRKY gene duplication events identified in the* P. trichocarpa* genome [61], with 29 out of 42 PtWRKY genes arising from segmental duplication. This comparison suggests that the expansion of LjWRKY genes is not necessarily dependent on gene duplication events.

We found few duplicated LjWRKY genes in the* L. japonicus* genome and there are at least three possible explanations: (1) most duplicated genes have been lost after segmental duplication events in LjWRKY genes [65]; (2) LjWRKY genes that are nonfunctional or duplicate the function of other copies are inclined to disappear to avoid fitness cost; and (3) the draft sequenced genome in* L. japonicus* may not yet contain all WRKY genes present in the genome.

The WRKY gene sequences contain two types of conserved introns (R-type and V-type) [22], and we found them in this study. In* Arabidopsis* [2] and* C. sativus* [59] subgroup IIa and IIb WRKY genes, R-type introns are inserted in domains located at the fourth amino acid (K residue) after the second C residue in the zinc finger region and there are no V-type introns. These results suggest that introns in subgroup IIa and IIb WRKY genes may have different origins.

The average length of the conserved introns (597 bp) in* L*.* japonicus* WRKY domains was longer than that in* Arabidopsis* (241 bp) but shorter than that in* M. truncatula* (705 bp). In nematodes and mammals, there is a dramatic decline in average intron size when there is increased gene expression [66]. However, the correlation between expression of WRKY genes in various plants and the intron length needs to be tested.

We found an interesting phenomenon which may provide context to interpret group I, II, and III WRKY gene origin and evolutionary relationships. Two conserved motifs (motif4 and/or motif9) were observed after motif1 (contains the conserved sequence WRKYGQK) in the N-terminal region, while, in the C-terminal region, other conserved motifs (motif5 or/and motif13) occur before motif1 (Figure 5). In addition, analysis of group II and III LjWRKY gene motifs revealed that most LjWRKY genes contain motif1, motif5, and/or motif13. However, motif1, motif4, and/or motif9 in the N-terminal region of group I genes are distributed in LjWRKY19, LjWRKY37, (group II) and LjWRKY25 and LjWRKY27 (group III), respectively (Figure 5). Previous research showed that group II and III WRKY genes evolved from group I through the loss of the N-terminal WRKY domain [2, 5]. However, our results indicate that some WRKY genes in groups II and III may have originated from the N-terminal region of group I. From the phylogenetic relationship, we found that LjWRK19 and LjWRKY37 (group II) clustered with group In with well-supported bootstrap values, suggesting that they have a common origin or ancestry. Moreover, we consider that some group III WRKY genes could have resulted from a mutation in the zinc finger motif in the N-terminal of group I genes. To confirm our inference, we analyzed the conserved motifs in MtWRKY genes. The same phenomenon was detected in MtWRKY genes based on analysis of location of motifs (data not shown) and 13 MtWRKY genes might have evolved from N-terminal of group I genes.

The natural selection pressure imposed by pathogens is expected to be diverse in different plants [67] and WRKY genes and NBS-LRR genes forming a fused gene may effectively resist a wide variety of pathogens. Fusion genes contain the C-terminal WRKY motif and a NBS-LRR (nucleotide-binding site-leucine-rich repeat) motif in the *R* gene was identified in AtWRKY [68, 69] and OsWRKY [22] genes. The *R* gene is mainly involved in a pathogen response pathway in plants [70]. Fusion gene, one gene included WRKY and NBS-LRR domains and/or motifs, was not detected in* L. japonicus*.

The majority of group III AtWRKY genes under positive selection are expressed in response to various abiotic stresses [59]. In contrast, the expression of group III CsWRKY genes shows that these genes are under purifying selection and are specialized to respond as single type of stress [59]. Ling et al. showed that positive selection may have resulted in the functional divergence of duplicated genes during the expansion of group III WRKY genes in* Arabidopsis* [59]. Similarly, our selection pressure study of group III LjWRKY and MtWRKY genes found that expansion of these genes may be under purifying selection, although gene duplication events occurred within these genes. Purifying selection may generate genes with conserved functions or pseudogenization [71] in duplicated group III MtWRKY genes. Therefore, we speculate that group III LjWRKY and MtWRKY genes may be more conservative in their response to stress.

## Figures and Tables

**Table 1:** WRKY family genes in *Lotus japonicus* genome.

Annotation ID^a^	Gene name	Group	Chr	Location	Annotation ID^a^	Gene name	Group	Chr	Location
CM0012.480.r2.a	LjWRKY1	I	1	58,226,335	*CM0432.2010.r2.d *	*LjWRKY37 *	*IIx *	*4 *	*7,529,554 *
CM0032.140.r2.d	LjWRKY2	IIc	1	17,061,228	CM0536.110.r2.d	LjWRKY38	IId	4	13,585,366
CM0032.170.r2.d	LjWRKY3	IIc	1	17,093,440	CM1622.200.r2.a	LjWRKY39	IIc	4	37,600,439
CM0105.740.r2.a	LjWRKY4	III	1	65,847,050	*CM0040.550.r2.d *	*LjWRKY40 *	*IIa *	*5 *	*5,190,496 *
CM0122.1300.r2.m	LjWRKY5	IIb	1	62,472,860	*CM0040.670.r2.d *	*LjWRKY41 *	*IIa *	*5 *	*5,268,779 *
CM0122.2160.r2.d	LjWRKY6	IIe	1	63,001,936	CM0239.70.r2.d	LjWRKY42	IIc	5	25,979,555
CM0123.240.r2.m	LjWRKY7	IIb	1	6,329,403	CM0852.250.r2.d	LjWRKY43	IId	5	2,801,268
CM0133.780.r2.m	LjWRKY8	IIc	1	23,799,861	CM1574.880.r2.m	LjWRKY44	IId	5	18,726,557
CM0147.250.r2.m	LjWRKY9	IIc	1	42,532,993	CM0037.660.r2.m	LjWRKY45	I	6	14,396,956
CM0315.160.r2.m	LjWRKY10	IIb	1	35,572,836	CM1034.270.r2.d	LjWRKY46	IIe	6	19,990,091
CM0320.110.r2.d	LjWRKY11	III	1	15,934,171	LjT11E14.30.r2.m	LjWRKY47	I	6	9,459,959
CM0433.90.r2.d	LjWRKY12	IIa	1	24,711,963	LjT46F23.30.r2.d	LjWRKY48	IId	6	17,672,968
*CM1413.480.r2.d *	*LjWRKY13 *	*I *	*1 *	*34,039,015 *	*LjSGA_012799.2 *	*LjWRKY49 *	*IIc *	*nd *	*nd *
CM1413.70.r2.d	LjWRKY14	IIe	1	33,660,455	*LjSGA_014003.1 *	*LjWRKY50 *	*I *	*nd *	*nd *
CM0058.50.r2.m	LjWRKY15	I	2	34,643,392	*LjSGA_015261.1 *	*LjWRKY51 *	*I *	*nd *	*nd *
CM0060.250.r2.a	LjWRKY16	III	2	31,851,611	LjSGA_020468.1	LjWRKY52	I	nd	nd
CM0120.570.r2.d	LjWRKY17	IIc	2	20,356,545	LjSGA_021425.1	LjWRKY53	III	nd	nd
CM0191.300.r2.a	LjWRKY18	I	2	43,680,266	LjSGA_025444.2	LjWRKY54	IIe	nd	nd
CM0608.50.r2.m	LjWRKY19	IIx	2	20,505,843	LjSGA_038153.1	LjWRKY55	nd	nd	nd
CM0608.70.r2.m	LjWRKY20	IIc	2	20,514,487	LjSGA_038411.1	LjWRKY56	nd	nd	nd
CM1895.20.r2.m	LjWRKY21	III	2	34,148,852	LjSGA_046833.1	LjWRKY57	IIa	nd	nd
LjB06N21.120.r2.a	LjWRKY22	IIb	2	42,171,450	LjSGA_050544.1	LjWRKY58	nd	nd	nd
CM0243.470.r2.m	LjWRKY23	IIb	3	39,562,863	LjSGA_055568.1	LjWRKY59	IIe	nd	nd
CM0936.60.r2.d	LjWRKY24	I	3	18,082,486	*LjSGA_058050.1 *	*LjWRKY60 *	*IIe *	*nd *	*nd *
LjT41F16.60.r2.d	LjWRKY25	III	3	33,152,607	LjSGA_058052.1	LjWRKY61	nd	nd	nd
LjT45M09.130.r2.d	LjWRKY26	IIa	3	474,491	LjSGA_066036.1	LjWRKY62	nd	nd	nd
CM0004.1290.r2.m	LjWRKY27	III	4	40,133,355	*LjSGA_069579.1 *	*LjWRKY63 *	*IIc *	*nd *	*nd *
CM0004.880.r2.d	LjWRKY28	IIe	4	39,837,881	LjSGA_074792.1	LjWRKY64	IIe	nd	nd
CM0007.1120.r2.a	LjWRKY29	IIc	4	3,358,814	LjSGA_084767.1	LjWRKY65	nd	nd	nd
CM0046.1360.r2.d	LjWRKY30	IIe	4	36,250,655	LjSGA_086809.1	LjWRKY66	nd	nd	nd
CM0179.130.r2.m	LjWRKY31	I	4	28,951,362	LjSGA_091481.1	LjWRKY67	nd	nd	nd
CM0227.580.r2.m	LjWRKY32	I	4	10,519,027	LjSGA_107504.1	LjWRKY68	nd	nd	nd
CM0244.860.r2.m	LjWRKY33	IId	4	30,871,468	LjSGA_116672.1	LjWRKY69	nd	nd	nd
CM0333.340.r2.d	LjWRKY34	IIc	4	32,856,509	LjT10P12.70.r2.d	LjWRKY70	IIb	nd	nd
CM0333.510.r2.a	LjWRKY35	IIc	4	32,985,615	LjT45D24.80.r2.d	LjWRKY71	IIb	nd	nd
CM0337.220.r2.d	LjWRKY36	IIb	4	3,852,684					

^a^Annotation IDs are from miyakogusa website http://www.kazusa.or.jp/lotus/.

Italics indicated pseudogenes.

Chr: chromosome.

nd: nondetermined.

LjWRKY, WRKY genes of *Lotus japonicas*.

**Table 2:** The number of WRKY gene in eight plants.

	Group I	Group IIa	Group IIb	Group IIc	Group IId	Group IIe	Group IIx	Group III	Total	Genome size (Mb)
LjWRKY	12	5	8	13	5	9	2	7	61	472
MtWRKY	19	6	8	19	8	8	0	13	81	375
AtWRKY^a^	13	4	7	18	7	9	0	14	72	125
OsWRKY^a^	15	4	8	15	7	11	0	36	96	480
CsWRKY^a^	10	4	4	16	8	7	0	6	55	367
VvWRKY^a^	12	4	7	14	6	7	0	5	55	487
BdWRKY^b^	17	3	6	21	6	10	0	23	86	272
PtWRKY^c^	50	5	9	13	13	4	0	10	104	485

^a^Data from Ling et al. (2011) [59]; ^b^data from Tripathi et al. (2012) [64]; ^c^data from He et al. (2012) [61].

**Table 3:** Consensus sequences of WRKY motif in *Lotus japonicus* proteins.

Motif	Width	Consensus sequences^a^
Motif1	29	ILDDGYRWRKYGQKVIKGNPYPRSYYRCT
Motif2	29	PGCPVKKHVERSAEDPSMVITTYEGEHNH
Motif3	21	WRKYGQKVVKGSPFPRSYYKC
Motif4	21	RSLDGHVTEITYKGxHNCPKP
Motif5	15	VREPRVAVQTKSEVD
Motif6	28	LVEAMAAALTADPNFTAALAAAISSIIG
Motif7	39	EELERLNAENKKLREMLDQMNENYNALQMHLVELMQKQK
Motif8	26	PAAMAMASTTSAAASMLLSGSMPSAD
Motif9	11	HPNCPAKKKVE
Motif10	113	QNLQVQNFQNQNVQQAAPSFSTTPSFNAATPVPPISYRNAIIPLPFNVANPAPNTH FNTSSFGSFLQGINGVDFVQSSRFMANNNQGLLRNNGLLQDMFVPAHMEFGGGGGRR
Motif11	24	CSASMATLSASAPFPTITLDLTQS
Motif12	42	DITEIISAPTSVTNSPILDLDLLDNVELDSNFPFNTPELFS
Motif13	15	KKRKMRVKRTVRVPA
Motif14	91	MPPPPPSPPLPPLSPLPLPPFHQQTSSFGSFKDLLTIEDFDPALFD WNPTTTTNTAAAADVTSPPLFTTQISHPVPSPATSNILPEDSFDV
Motif15	27	SGGGELDDDEPDAKRWKGEGENDGYSA
Motif16	15	LLLNRTGHARFRRAP
Motif17	26	MEHLHKWEQKALINELIQGMELARKL
Motif18	14	MDDDWDLHAVVRGC
Motif19	19	MSVMPEFLQQGLNSSPVNV
Motif20	39	QCSGDIEGELTGEAMELGGEKAIESARALLSIGFEIKPC

^a^Consensus amino acid sequence was derived from MEME program.

**Table 4:** Likelihood ratio test results of group III *Lotus japonicus* WEKY and *Medicago truncatula* WRKY genes.

Node^a^	*d*_N_/*d*_S_ under M0^b^	2Δln⁡*L* M3 versus M0	2Δln⁡*L* M8 versus M7	M8 estimates^c^	Number of positive selection site^d^
Group III LjWRKY					
1	0.29349	67.57**	0.000074	*ω* = 1 (*P* = 0.72, *q* = 1.2)	0
2	0.29349	67.57**	0.000074	*ω* = 1 (*P* = 0.72, *q* = 1.22)	0
3	0.25749	74.47**	0.095776	*ω* = 1.13 (*P* = 0.84, *q* = 2)	0
4	0.23855	112.17**	0.000198	*ω* = 1 (*P* = 0.8, *q* = 1.8)	0

Group III MtWRKY					
1	0.31282	3.45	0.000016	*ω* = 1 (*P* = 0.77, *q* = 1.44)	0
2	0.28717	4.31	0.000042	*ω* = 1 (*P* = 1.9, *q* = 4.18)	0
3	0.27266	27.39**	0.178716	*ω* = 2.19 (*P* = 0.52, *q* = 0.93)	1
4	0.26004	70.18**	0.000078	*ω* = 1 (*P* = 0.76, *q* = 1.51)	0
5	0.24433	81.88**	0.000204	*ω* = 1 (*P* = 0.74, *q* = 1.48)	0
6	0.00517	43.43**	0.029352	*ω* = 34.48 (*P* = 1.09, *q* = 61.42)	0
7	0.20188	209.83**	0.000302	*ω* = 1 (*P* = 0.64, *q* = 1.68)	0
8	0.18564	233.88**	0.000154	*ω* = 2.5 (*P* = 0.6, *q* = 1.8)	0

*P < 0.05 and **P < 0.01 (*χ* ^2^ test).

^a^Node number from the phylogenetic tree.

^b^ *d*_N_/*d*_S_ is the average ratio over sites under a codon model with one ratio.

^c^ *ω* was estimated under model M8; *P* and *q* are the parameters of the beta distribution.

^d^The number of amino acid sites estimated to have undergone positive selection under M8.
